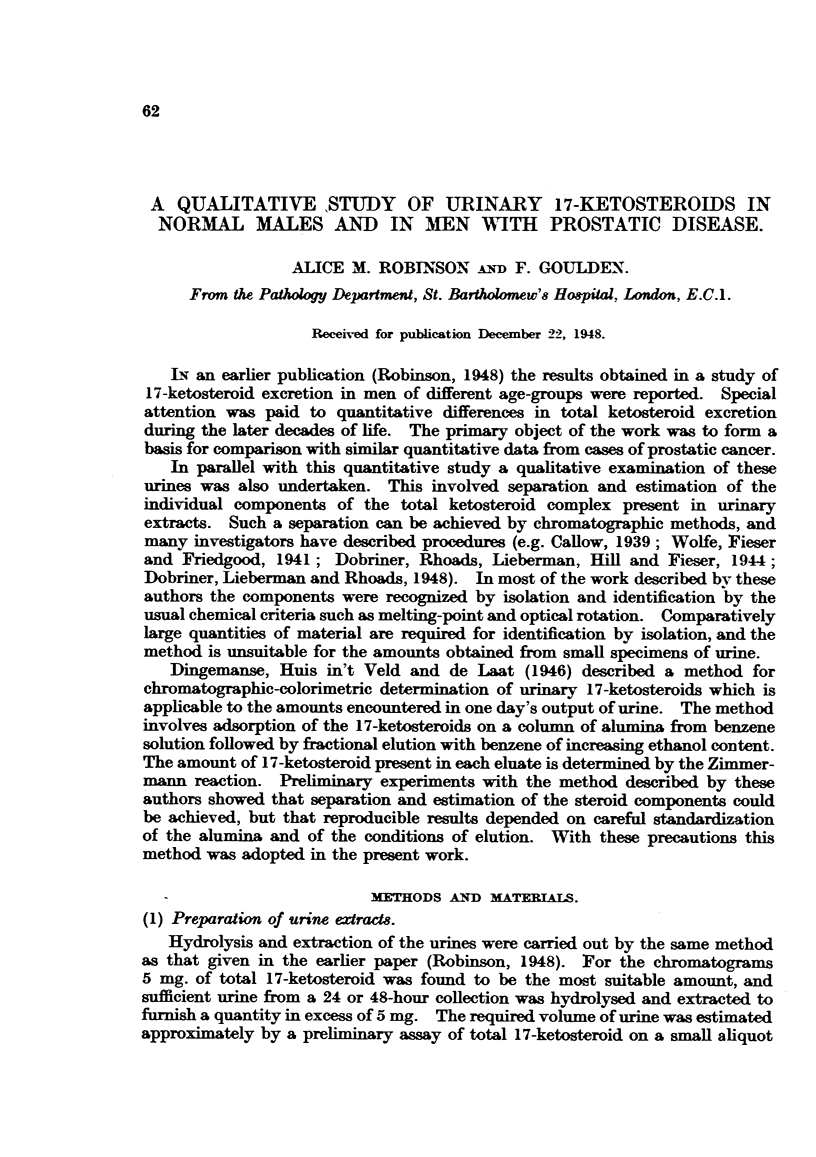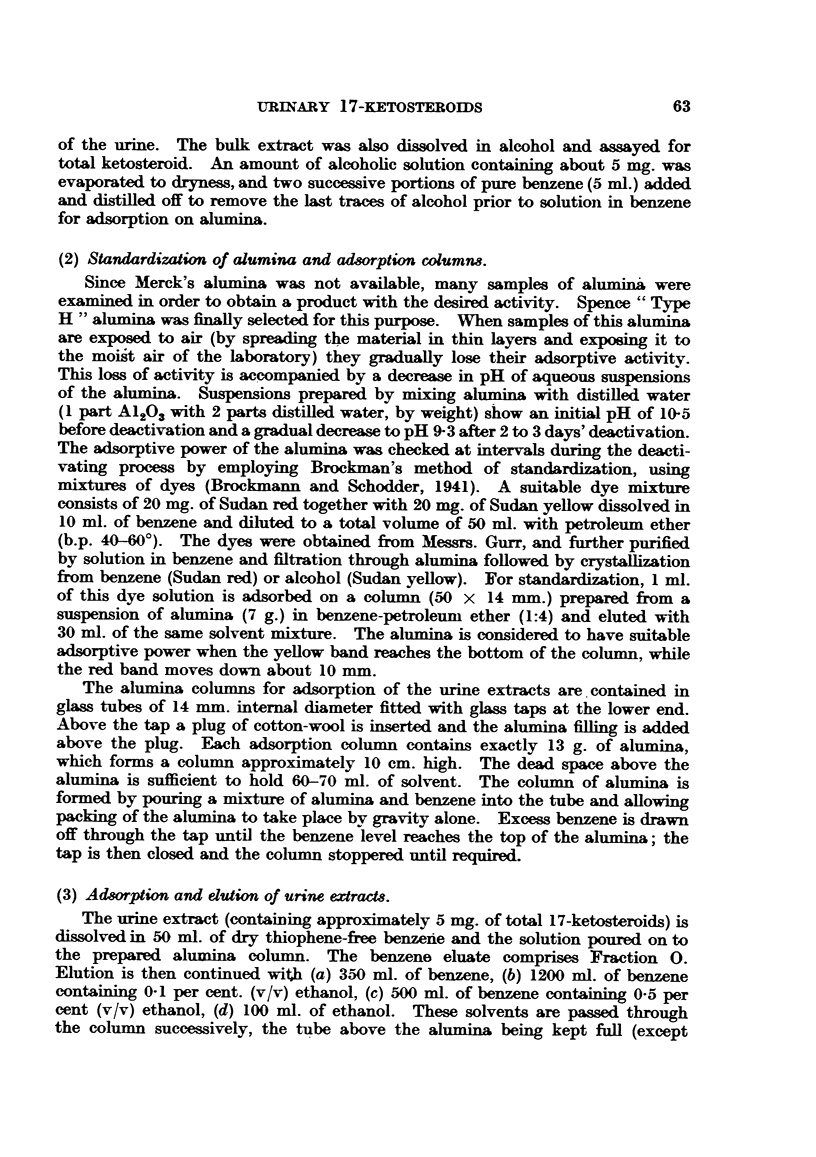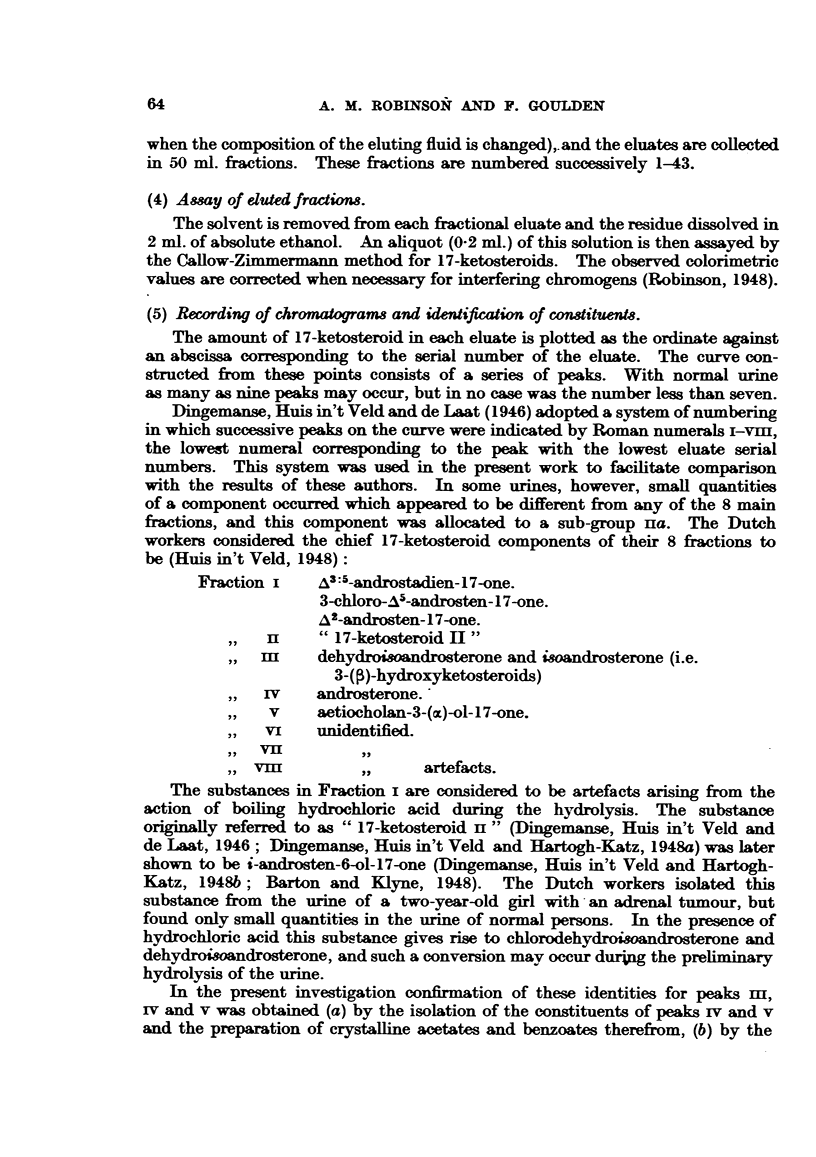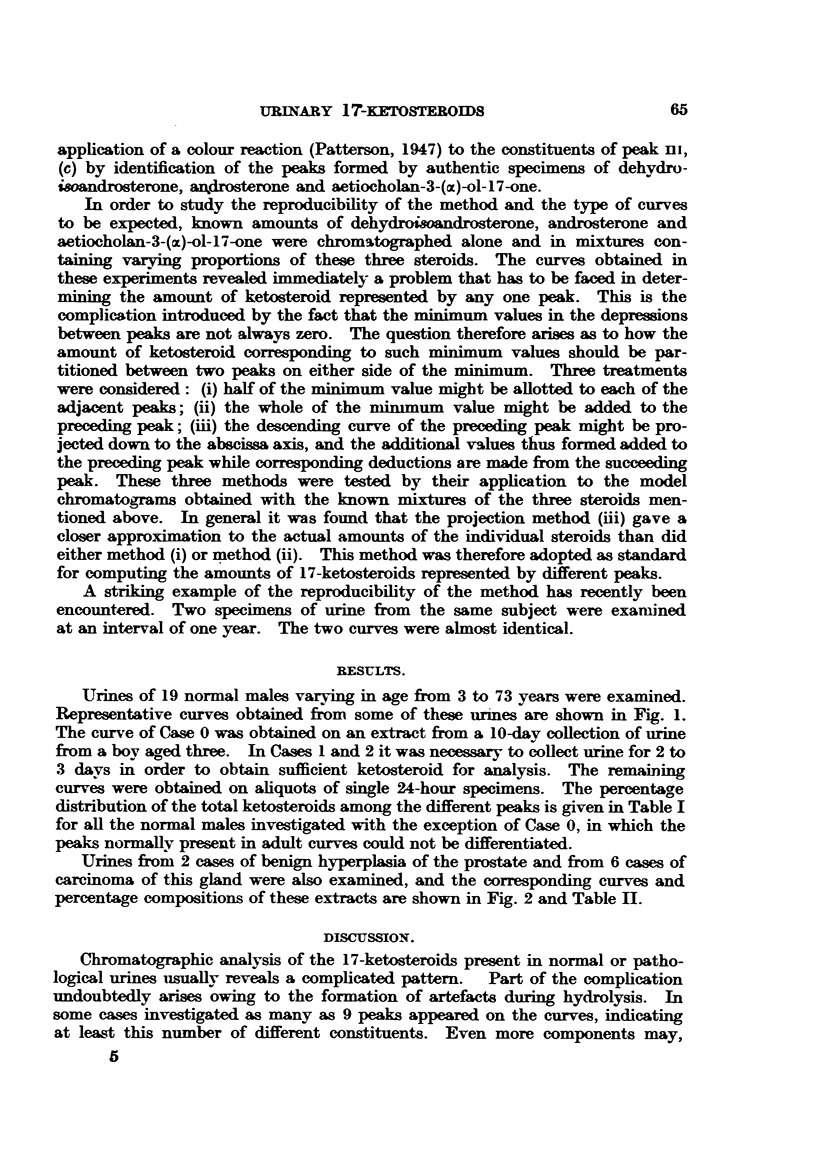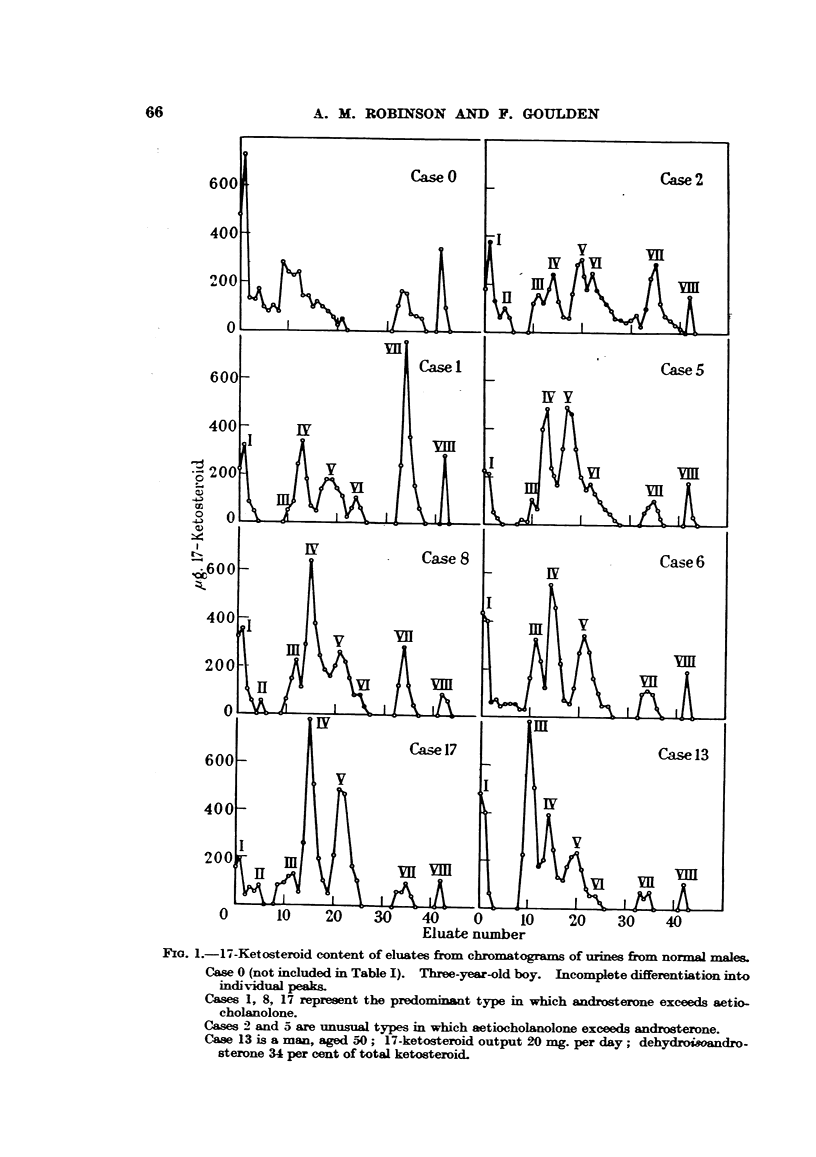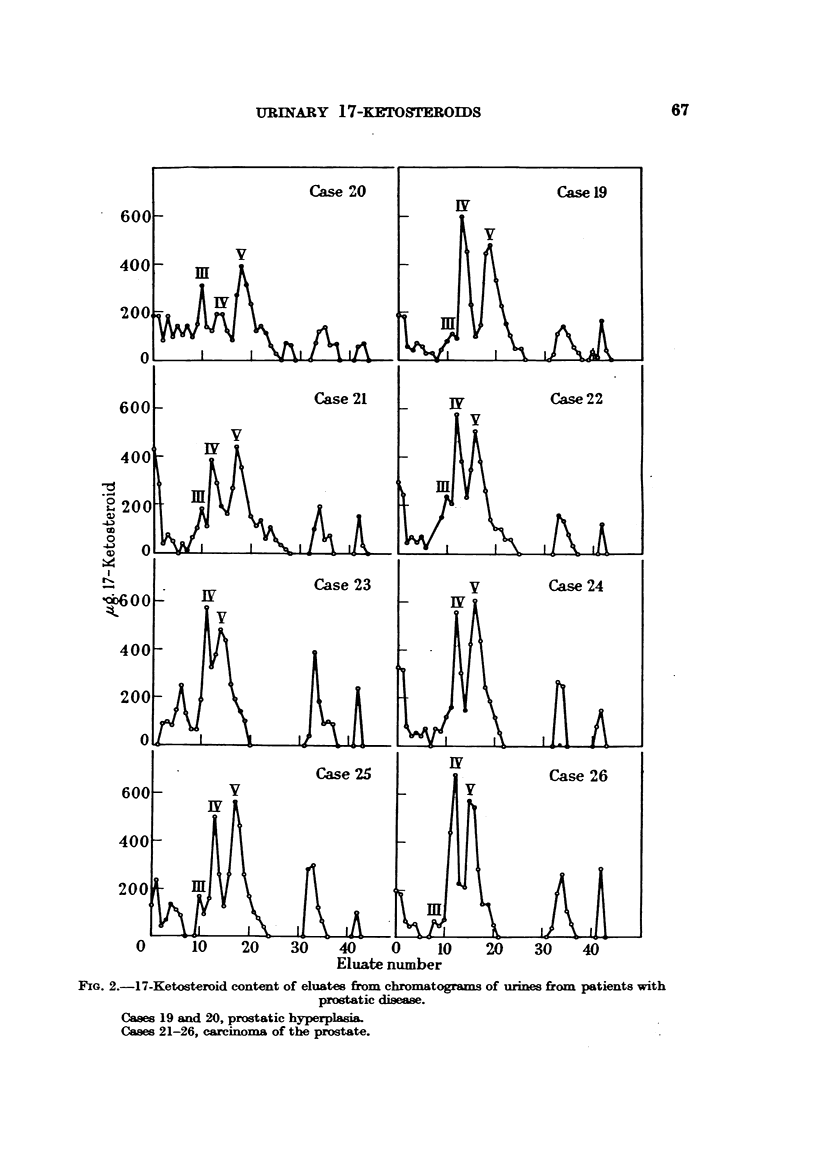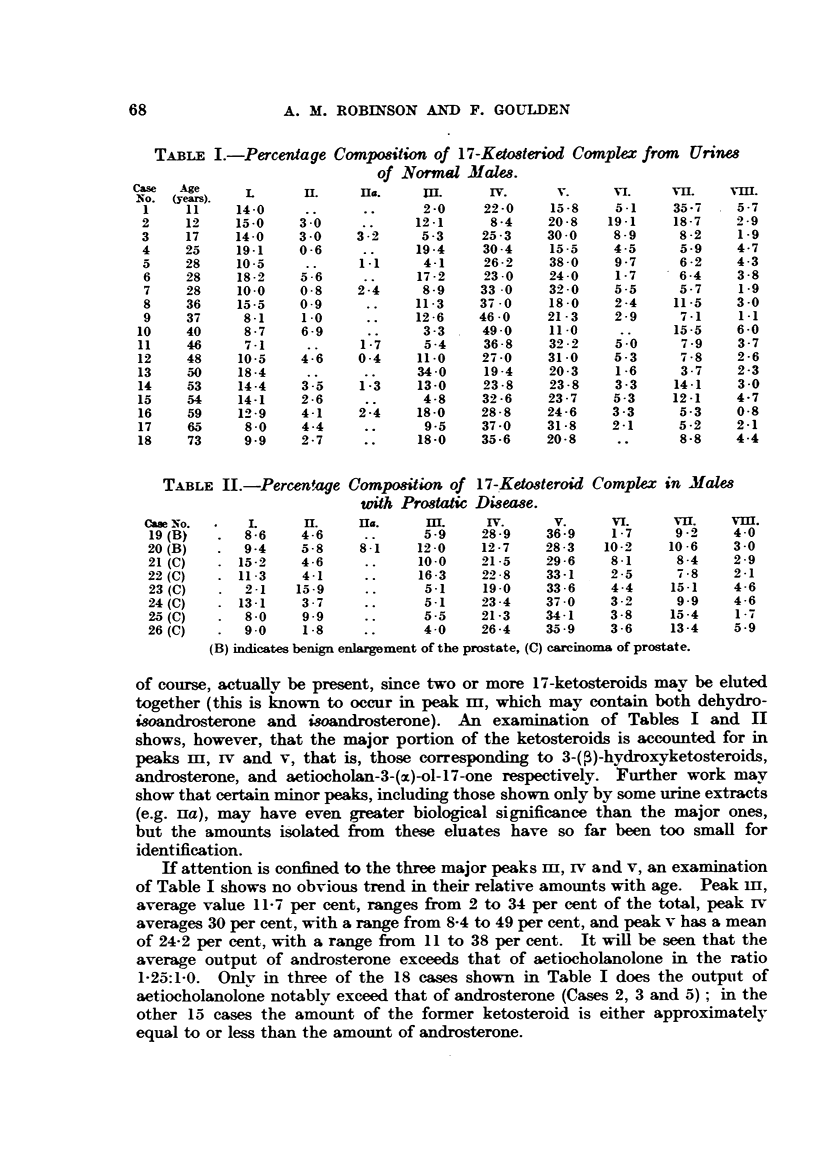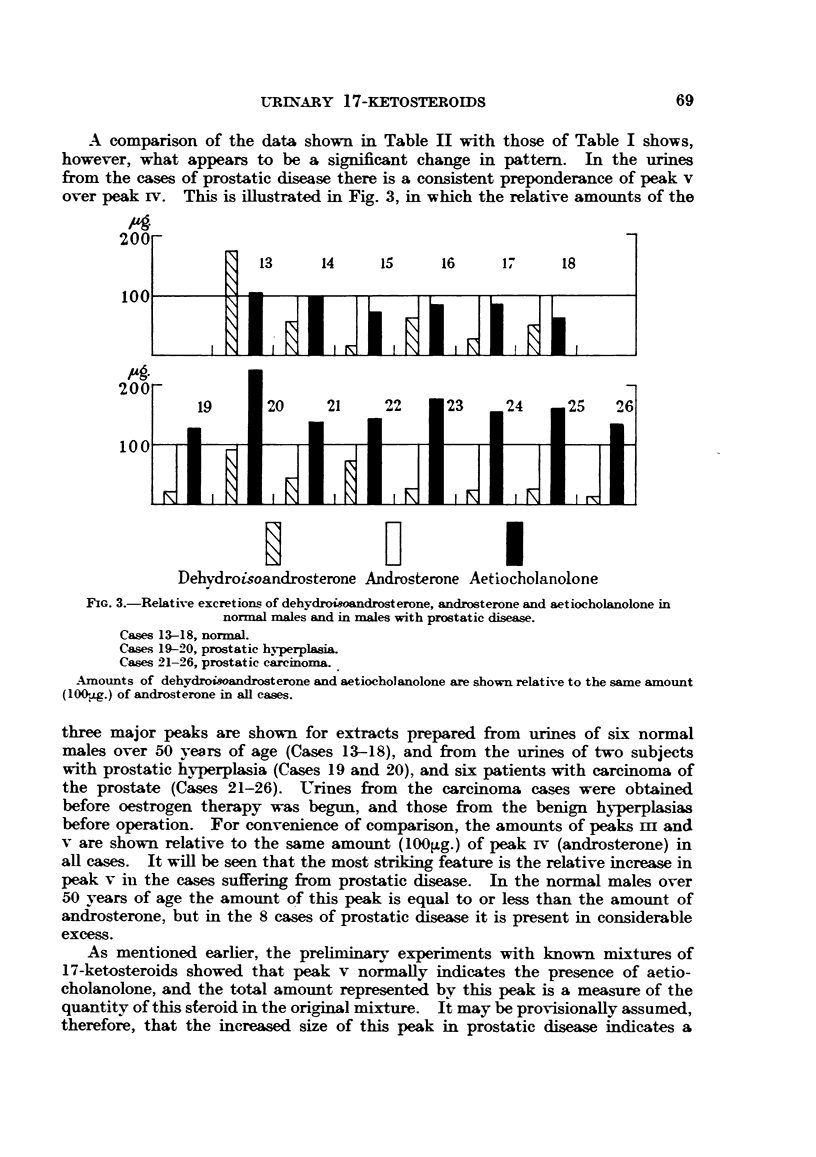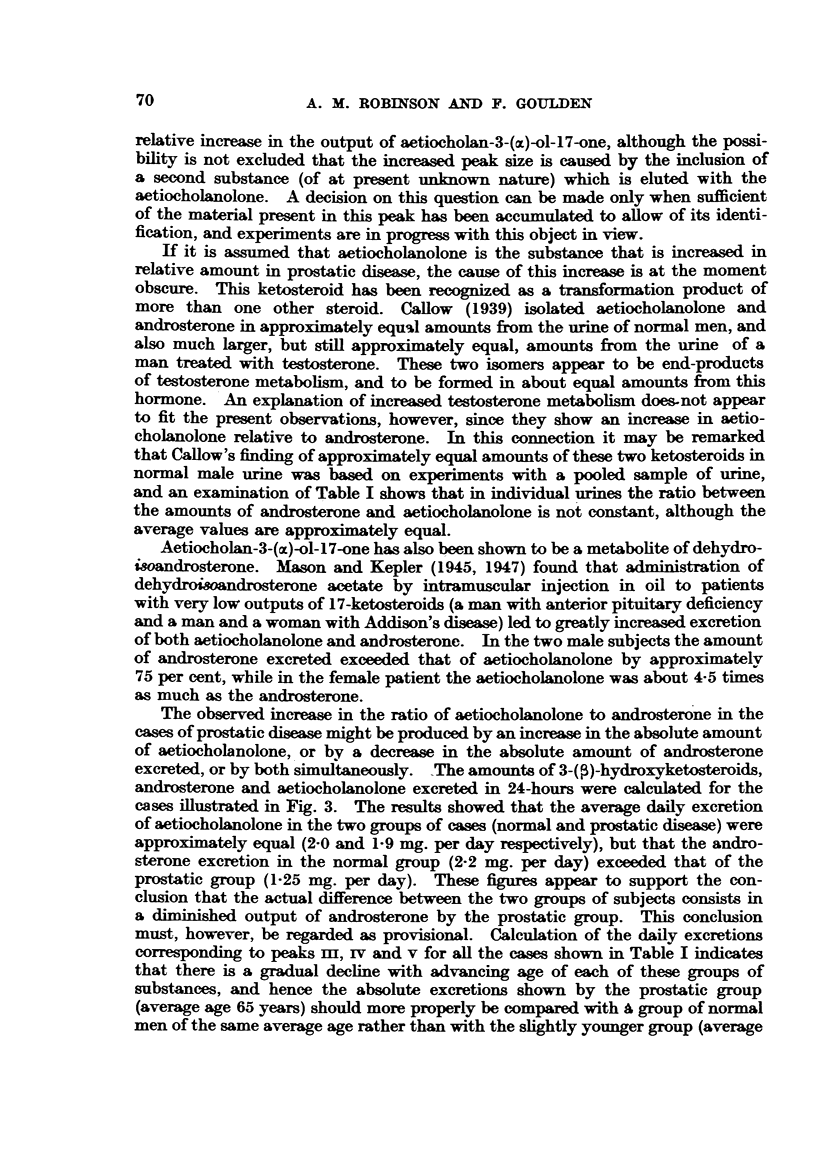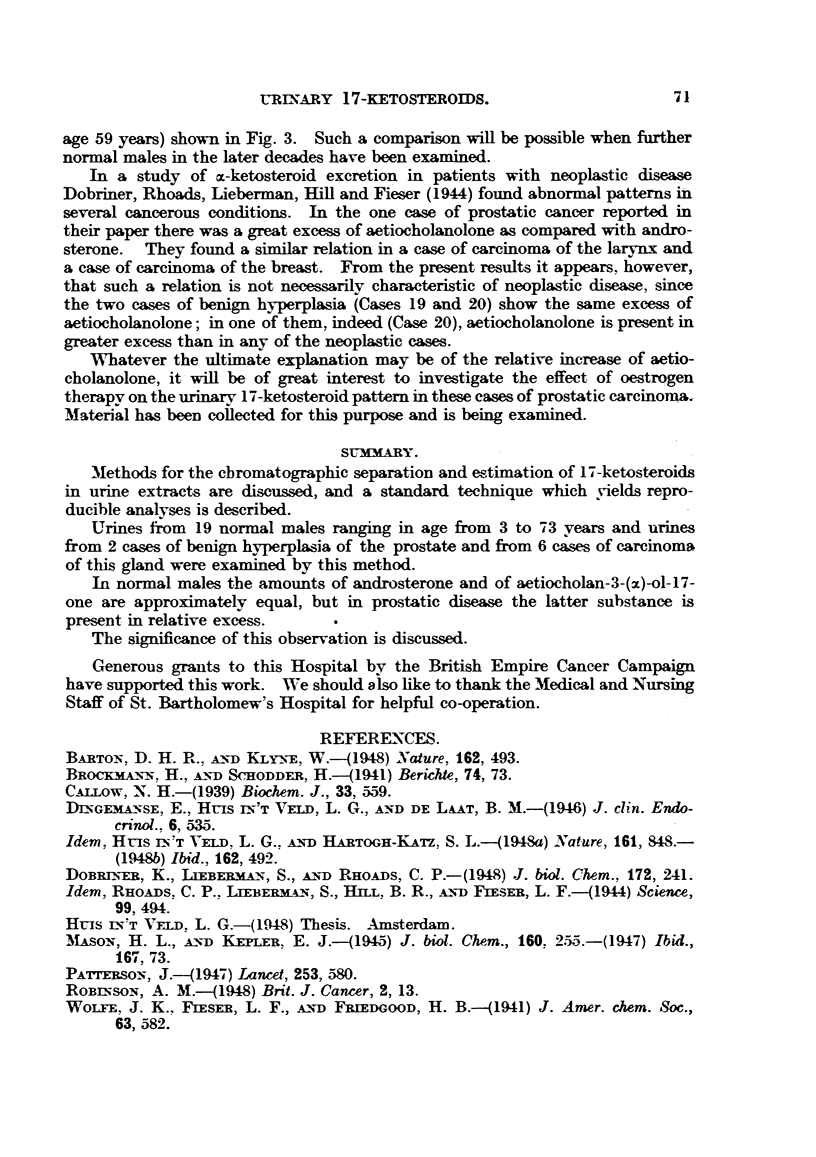# A Qualitative Study of Urinary 17-Ketosteroids in Normal Males and in Men with Prostatic Disease

**DOI:** 10.1038/bjc.1949.7

**Published:** 1949-03

**Authors:** Alice M. Robinson, F. Goulden


### A QUALITATIVE STUDY OF URINARY 17-KETOSTEROIDS IN NORMAL MALES AND IN MEN WITH PROSTATIC DISEASE.

ALICE M. ROBINSON AND F. GOULDEN.

*From the Pathology Department, St. Bartholomew's Hospital, London, E.C.*1.

Received for publication December 22, 1948.

In an earlier publication (Robinson, 1948) the results obtained in a study of 17-ketosteroid excretion in men of different age-groups were reported. Special attention was paid to quantitative differences in total ketosteroid excretion during the later decades of life. The primary object of the work was to form a basis for comparison with similar quantitative data from cases of prostatic cancer.

In parallel with this quantitative study a qualitative examination of these urines was also undertaken. This involved separation and estimation of the individual components of the total ketosteroid complex present in urinary extracts. Such a separation can be achieved by chromatographic methods, and many investigators have described procedures (e.g. Callow, 1939 ; Wolfe, Fieser and Friedgood, 1941 ; Dobriner, Rhoads, Lieberman, Hill and Fieser, 1944 ; Dobriner, Lieberman and Rhoads, 1948). In most of the work described by these authors the components were recognized by isolation and identification by the usual chemical criteria such as melting-point and optical rotation. Comparatively large quantities of material are required for identification by isolation, and the method is unsuitable for the amounts obtained from small specimens of urine.

Dingemanse, Huis in't Veld and de Laat (1946) described a method for chromatographic-colorimetric determination of urinary 17-ketosteroids which is applicable to the amounts encountered in one day's output of urine. The method involves adsorption of the 17-ketosteroids on a column of alumina from benzene solution followed by fractional elution with benzene of increasing ethanol content. The amount of 17-ketosteroid present in each eluate is determined by the Zimmermann reaction. Preliminary experiments with the method described by these authors showed that separation and estimation of the steroid components could be achieved, but that reproducible results depended on careful standardization of the alumina and of the conditions of elution. With these precautions this method was adopted in the present work.

### METHODS AND MATERIALS.

### (1) *Preparation of urine extracts.*

Hydrolysis and extraction of the urines were carried out by the same method as that given in the earlier paper (Robinson, 1948). For the chromatograms 5 mg. of total 17-ketosteroid was found to be the most suitable amount, and sufficient urine from a 24 or 48-hour collection was hydrolysed and extracted to furnish a quantity in excess of 5 mg. The required volume of urine was estimated approximately by a preliminary assay of total 17-ketosteroid on a small aliquot of the urine. The bulk extract was also dissolved in alcohol and assayed for total ketosteroid. An amount of alcoholic solution containing about 5 mg. was evaporated to dryness, and two successive portions of pure benzene (5 ml.) added and distilled off to remove the last traces of alcohol prior to solution in benzene for adsorption on alumina.

(2) *Standardization of alumina and adsorption columns.*

Since Merck's alumina was not available, many samples of alumina were examined in order to obtain a product with the desired activity. Spence " Type H " alumina was finally selected for this purpose. When samples of this alumina are exposed to air (by spreading the material in thin layers and exposing it to the moist air of the laboratory) they gradually lose their adsorptive activity. This loss of activity is accompanied by a decrease in pH of aqueous suspensions of the alumina. Suspensions prepared by mixing alumina with distilled water (1 part $Al_2O_3$ with 2 parts distilled water, by weight) show an initial pH of 10·5 before deactivation and a gradual decrease to pH 9·3 after 2 to 3 days' deactivation. The adsorptive power of the alumina was checked at intervals during the deactivating process by employing Brockman's method of standardization, using mixtures of dyes (Brockmann and Schodder, 1941). A suitable dye mixture consists of 20 mg. of Sudan red together with 20 mg. of Sudan yellow dissolved in 10 ml. of benzene and diluted to a total volume of 50 ml. with petroleum ether (b.p. 40–60°). The dyes were obtained from Messrs. Gurr, and further purified by solution in benzene and filtration through alumina followed by crystallization from benzene (Sudan red) or alcohol (Sudan yellow). For standardization, 1 ml. of this dye solution is adsorbed on a column (50 × 14 mm.) prepared from a suspension of alumina (7 g.) in benzene-petroleum ether (1:4) and eluted with 30 ml. of the same solvent mixture. The alumina is considered to have suitable adsorptive power when the yellow band reaches the bottom of the column, while the red band moves down about 10 mm.

The alumina columns for adsorption of the urine extracts are contained in glass tubes of 14 mm. internal diameter fitted with glass taps at the lower end. Above the tap a plug of cotton-wool is inserted and the alumina filling is added above the plug. Each adsorption column contains exactly 13 g. of alumina, which forms a column approximately 10 cm. high. The dead space above the alumina is sufficient to hold 60–70 ml. of solvent. The column of alumina is formed by pouring a mixture of alumina and benzene into the tube and allowing packing of the alumina to take place by gravity alone. Excess benzene is drawn off through the tap until the benzene level reaches the top of the alumina; the tap is then closed and the column stoppered until required.

(3) *Adsorption and elution of urine extracts.*

The urine extract (containing approximately 5 mg. of total 17-ketosteroids) is dissolved in 50 ml. of dry thiophene-free benzene and the solution poured on to the prepared alumina column. The benzene eluate comprises Fraction O. Elution is then continued with (*a*) 350 ml. of benzene, (*b*) 1200 ml. of benzene containing 0·1 per cent. (v/v) ethanol, (*c*) 500 ml. of benzene containing 0·5 per cent (v/v) ethanol, (*d*) 100 ml. of ethanol. These solvents are passed through the column successively, the tube above the alumina being kept full (except when the composition of the eluting fluid is changed), and the eluates are collected in 50 ml. fractions. These fractions are numbered successively 1–43.

(4) *Assay of eluted fractions.*

The solvent is removed from each fractional eluate and the residue dissolved in 2 ml. of absolute ethanol. An aliquot (0·2 ml.) of this solution is then assayed by the Callow-Zimmermann method for 17-ketosteroids. The observed colorimetric values are corrected when necessary for interfering chromogens (Robinson, 1948).

(5) *Recording of chromatograms and identification of constituents.*

The amount of 17-ketosteroid in each eluate is plotted as the ordinate against an abscissa corresponding to the serial number of the eluate. The curve constructed from these points consists of a series of peaks. With normal urine as many as nine peaks may occur, but in no case was the number less than seven.

Dingemanse, Huis in't Veld and de Laat (1946) adopted a system of numbering in which successive peaks on the curve were indicated by Roman numerals I–VIII, the lowest numeral corresponding to the peak with the lowest eluate serial numbers. This system was used in the present work to facilitate comparison with the results of these authors. In some urines, however, small quantities of a component occurred which appeared to be different from any of the 8 main fractions, and this component was allocated to a sub-group II*a*. The Dutch workers considered the chief 17-ketosteroid components of their 8 fractions to be (Huis in't Veld, 1948):

| | |
|---|---|
| Fraction I | $\Delta^{3:5}$-androstadien-17-one. |
| | 3-chloro-$\Delta^{5}$-androsten-17-one. |
| | $\Delta^{2}$-androsten-17-one. |
| ,, II | " 17-ketosteroid II " |
| ,, III | dehydro*iso*androsterone and *iso*androsterone (i.e. 3-(β)-hydroxyketosteroids) |
| ,, IV | androsterone. |
| ,, V | aetiocholan-3-(α)-ol-17-one. |
| ,, VI | unidentified. |
| ,, VII | ,, |
| ,, VIII | ,, artefacts. |

The substances in Fraction I are considered to be artefacts arising from the action of boiling hydrochloric acid during the hydrolysis. The substance originally referred to as " 17-ketosteroid II " (Dingemanse, Huis in't Veld and de Laat, 1946; Dingemanse, Huis in't Veld and Hartogh-Katz, 1948*a*) was later shown to be *i*-androsten-6-ol-17-one (Dingemanse, Huis in't Veld and Hartogh-Katz, 1948*b*; Barton and Klyne, 1948). The Dutch workers isolated this substance from the urine of a two-year-old girl with an adrenal tumour, but found only small quantities in the urine of normal persons. In the presence of hydrochloric acid this substance gives rise to chlorodehydro*iso*androsterone and dehydro*iso*androsterone, and such a conversion may occur during the preliminary hydrolysis of the urine.

In the present investigation confirmation of these identities for peaks III, IV and V was obtained (*a*) by the isolation of the constituents of peaks IV and V and the preparation of crystalline acetates and benzoates therefrom, (*b*) by the application of a colour reaction (Patterson, 1947) to the constituents of peak III, (*c*) by identification of the peaks formed by authentic specimens of dehydro*iso*androsterone, androsterone and aetiocholan-3-(α)-ol-17-one.

In order to study the reproducibility of the method and the type of curves to be expected, known amounts of dehydro*iso*androsterone, androsterone and aetiocholan-3-(α)-ol-17-one were chromatographed alone and in mixtures containing varying proportions of these three steroids. The curves obtained in these experiments revealed immediately a problem that has to be faced in determining the amount of ketosteroid represented by any one peak. This is the complication introduced by the fact that the minimum values in the depressions between peaks are not always zero. The question therefore arises as to how the amount of ketosteroid corresponding to such minimum values should be partitioned between two peaks on either side of the minimum. Three treatments were considered: (i) half of the minimum value might be allotted to each of the adjacent peaks; (ii) the whole of the minimum value might be added to the preceding peak; (iii) the descending curve of the preceding peak might be projected down to the abscissa axis, and the additional values thus formed added to the preceding peak while corresponding deductions are made from the succeeding peak. These three methods were tested by their application to the model chromatograms obtained with the known mixtures of the three steroids mentioned above. In general it was found that the projection method (iii) gave a closer approximation to the actual amounts of the individual steroids than did either method (i) or method (ii). This method was therefore adopted as standard for computing the amounts of 17-ketosteroids represented by different peaks.

A striking example of the reproducibility of the method has recently been encountered. Two specimens of urine from the same subject were examined at an interval of one year. The two curves were almost identical.

### RESULTS.

Urines of 19 normal males varying in age from 3 to 73 years were examined. Representative curves obtained from some of these urines are shown in Fig. 1. The curve of Case 0 was obtained on an extract from a 10-day collection of urine from a boy aged three. In Cases 1 and 2 it was necessary to collect urine for 2 to 3 days in order to obtain sufficient ketosteroid for analysis. The remaining curves were obtained on aliquots of single 24-hour specimens. The percentage distribution of the total ketosteroids among the different peaks is given in Table I for all the normal males investigated with the exception of Case 0, in which the peaks normally present in adult curves could not be differentiated.

Urines from 2 cases of benign hyperplasia of the prostate and from 6 cases of carcinoma of this gland were also examined, and the corresponding curves and percentage compositions of these extracts are shown in Fig. 2 and Table II.

### DISCUSSION.

Chromatographic analysis of the 17-ketosteroids present in normal or pathological urines usually reveals a complicated pattern. Part of the complication undoubtedly arises owing to the formation of artefacts during hydrolysis. In some cases investigated as many as 9 peaks appeared on the curves, indicating at least this number of different constituents. Even more components may,

FIG. 1.—17-Ketosteroid content of eluates from chromatograms of urines from normal males.

Case 0 (not included in Table I). Three-year-old boy. Incomplete differentiation into individual peaks.

Cases 1, 8, 17 represent the predominant type in which androsterone exceeds aetiocholanolone.

Cases 2 and 5 are unusual types in which aetiocholanolone exceeds androsterone.

Case 13 is a man, aged 50; 17-ketosteroid output 20 mg. per day; dehydro*iso*androsterone 34 per cent of total ketosteroid.

FIG. 2.—17-Ketosteroid content of eluates from chromatograms of urines from patients with prostatic disease.

Cases 19 and 20, prostatic hyperplasia.
Cases 21–26, carcinoma of the prostate.

TABLE I.—*Percentage Composition of 17-Ketosteriod Complex from Urines of Normal Males.*

| Case No. | Age (years). | I. | II. | IIa. | III. | IV. | V. | VI. | VII. | VIII. |
|---|---|---|---|---|---|---|---|---|---|---|
| 1 | 11 | 14·0 | .. | .. | 2·0 | 22·0 | 15·8 | 5·1 | 35·7 | 5·7 |
| 2 | 12 | 15·0 | 3·0 | .. | 12·1 | 8·4 | 20·8 | 19·1 | 18·7 | 2·9 |
| 3 | 17 | 14·0 | 3·0 | 3·2 | 5·3 | 25·3 | 30·0 | 8·9 | 8·2 | 1·9 |
| 4 | 25 | 19·1 | 0·6 | .. | 19·4 | 30·4 | 15·5 | 4·5 | 5·9 | 4·7 |
| 5 | 28 | 10·5 | .. | 1·1 | 4·1 | 26·2 | 38·0 | 9·7 | 6·2 | 4·3 |
| 6 | 28 | 18·2 | 5·6 | .. | 17·2 | 23·0 | 24·0 | 1·7 | 6·4 | 3·8 |
| 7 | 28 | 10·0 | 0·8 | 2·4 | 8·9 | 33·0 | 32·0 | 5·5 | 5·7 | 1·9 |
| 8 | 36 | 15·5 | 0·9 | .. | 11·3 | 37·0 | 18·0 | 2·4 | 11·5 | 3·0 |
| 9 | 37 | 8·1 | 1·0 | .. | 12·6 | 46·0 | 21·3 | 2·9 | 7·1 | 1·1 |
| 10 | 40 | 8·7 | 6·9 | .. | 3·3 | 49·0 | 11·0 | .. | 15·5 | 6·0 |
| 11 | 46 | 7·1 | .. | 1·7 | 5·4 | 36·8 | 32·2 | 5·0 | 7·9 | 3·7 |
| 12 | 48 | 10·5 | 4·6 | 0·4 | 11·0 | 27·0 | 31·0 | 5·3 | 7·8 | 2·6 |
| 13 | 50 | 18·4 | .. | .. | 34·0 | 19·4 | 20·3 | 1·6 | 3·7 | 2·3 |
| 14 | 53 | 14·4 | 3·5 | 1·3 | 13·0 | 23·8 | 23·8 | 3·3 | 14·1 | 3·0 |
| 15 | 54 | 14·1 | 2·6 | .. | 4·8 | 32·6 | 23·7 | 5·3 | 12·1 | 4·7 |
| 16 | 59 | 12·9 | 4·1 | 2·4 | 18·0 | 28·8 | 24·6 | 3·3 | 5·3 | 0·8 |
| 17 | 65 | 8·0 | 4·4 | .. | 9·5 | 37·0 | 31·8 | 2·1 | 5·2 | 2·1 |
| 18 | 73 | 9·9 | 2·7 | .. | 18·0 | 35·6 | 20·8 | .. | 8·8 | 4·4 |

TABLE II.—*Percentage Composition of 17-Ketosteroid Complex in Males with Prostatic Disease.*

| Case No. | I. | II. | IIa. | III. | IV. | V. | VI. | VII. | VIII. |
|---|---|---|---|---|---|---|---|---|---|
| 19 (B) | 8·6 | 4·6 | .. | 5·9 | 28·9 | 36·9 | 1·7 | 9·2 | 4·0 |
| 20 (B) | 9·4 | 5·8 | 8·1 | 12·0 | 12·7 | 28·3 | 10·2 | 10·6 | 3·0 |
| 21 (C) | 15·2 | 4·6 | .. | 10·0 | 21·5 | 29·6 | 8·1 | 8·4 | 2·9 |
| 22 (C) | 11·3 | 4·1 | .. | 16·3 | 22·8 | 33·1 | 2·5 | 7·8 | 2·1 |
| 23 (C) | 2·1 | 15·9 | .. | 5·1 | 19·0 | 33·6 | 4·4 | 15·1 | 4·6 |
| 24 (C) | 13·1 | 3·7 | .. | 5·1 | 23·4 | 37·0 | 3·2 | 9·9 | 4·6 |
| 25 (C) | 8·0 | 9·9 | .. | 5·5 | 21·3 | 34·1 | 3·8 | 15·4 | 1·7 |
| 26 (C) | 9·0 | 1·8 | .. | 4·0 | 26·4 | 35·9 | 3·6 | 13·4 | 5·9 |

(B) indicates benign enlargement of the prostate, (C) carcinoma of prostate.

of course, actually be present, since two or more 17-ketosteroids may be eluted together (this is known to occur in peak III, which may contain both dehydro*iso*androsterone and *iso*androsterone). An examination of Tables I and II shows, however, that the major portion of the ketosteroids is accounted for in peaks III, IV and V, that is, those corresponding to 3-(β)-hydroxyketosteroids, androsterone, and aetiocholan-3-(α)-ol-17-one respectively. Further work may show that certain minor peaks, including those shown only by some urine extracts (e.g. II*a*), may have even greater biological significance than the major ones, but the amounts isolated from these eluates have so far been too small for identification.

If attention is confined to the three major peaks III, IV and V, an examination of Table I shows no obvious trend in their relative amounts with age. Peak III, average value 11·7 per cent, ranges from 2 to 34 per cent of the total, peak IV averages 30 per cent, with a range from 8·4 to 49 per cent, and peak V has a mean of 24·2 per cent, with a range from 11 to 38 per cent. It will be seen that the average output of androsterone exceeds that of aetiocholanolone in the ratio 1·25:1·0. Only in three of the 18 cases shown in Table I does the output of aetiocholanolone notably exceed that of androsterone (Cases 2, 3 and 5) ; in the other 15 cases the amount of the former ketosteroid is either approximately equal to or less than the amount of androsterone.

A comparison of the data shown in Table II with those of Table I shows, however, what appears to be a significant change in pattern. In the urines from the cases of prostatic disease there is a consistent preponderance of peak v over peak IV. This is illustrated in Fig. 3, in which the relative amounts of the

FIG. 3.—Relative excretions of dehydro*iso*androsterone, androsterone and aetiocholanolone in normal males and in males with prostatic disease.

Cases 13–18, normal.
Cases 19–20, prostatic hyperplasia.
Cases 21–26, prostatic carcinoma.

Amounts of dehydro*iso*androsterone and aetiocholanolone are shown relative to the same amount (100μg.) of androsterone in all cases.

three major peaks are shown for extracts prepared from urines of six normal males over 50 years of age (Cases 13–18), and from the urines of two subjects with prostatic hyperplasia (Cases 19 and 20), and six patients with carcinoma of the prostate (Cases 21–26). Urines from the carcinoma cases were obtained before oestrogen therapy was begun, and those from the benign hyperplasias before operation. For convenience of comparison, the amounts of peaks III and V are shown relative to the same amount (100μg.) of peak IV (androsterone) in all cases. It will be seen that the most striking feature is the relative increase in peak V in the cases suffering from prostatic disease. In the normal males over 50 years of age the amount of this peak is equal to or less than the amount of androsterone, but in the 8 cases of prostatic disease it is present in considerable excess.

As mentioned earlier, the preliminary experiments with known mixtures of 17-ketosteroids showed that peak V normally indicates the presence of aetiocholanolone, and the total amount represented by this peak is a measure of the quantity of this steroid in the original mixture. It may be provisionally assumed, therefore, that the increased size of this peak in prostatic disease indicates a relative increase in the output of aetiocholan-3-(α)-ol-17-one, although the possibility is not excluded that the increased peak size is caused by the inclusion of a second substance (of at present unknown nature) which is eluted with the aetiocholanolone. A decision on this question can be made only when sufficient of the material present in this peak has been accumulated to allow of its identification, and experiments are in progress with this object in view.

If it is assumed that aetiocholanolone is the substance that is increased in relative amount in prostatic disease, the cause of this increase is at the moment obscure. This ketosteroid has been recognized as a transformation product of more than one other steroid. Callow (1939) isolated aetiocholanolone and androsterone in approximately equal amounts from the urine of normal men, and also much larger, but still approximately equal, amounts from the urine of a man treated with testosterone. These two isomers appear to be end-products of testosterone metabolism, and to be formed in about equal amounts from this hormone. An explanation of increased testosterone metabolism does not appear to fit the present observations, however, since they show an increase in aetiocholanolone relative to androsterone. In this connection it may be remarked that Callow's finding of approximately equal amounts of these two ketosteroids in normal male urine was based on experiments with a pooled sample of urine, and an examination of Table I shows that in individual urines the ratio between the amounts of androsterone and aetiocholanolone is not constant, although the average values are approximately equal.

Aetiocholan-3-(α)-ol-17-one has also been shown to be a metabolite of dehydro*iso*androsterone. Mason and Kepler (1945, 1947) found that administration of dehydro*iso*androsterone acetate by intramuscular injection in oil to patients with very low outputs of 17-ketosteroids (a man with anterior pituitary deficiency and a man and a woman with Addison's disease) led to greatly increased excretion of both aetiocholanolone and androsterone. In the two male subjects the amount of androsterone excreted exceeded that of aetiocholanolone by approximately 75 per cent, while in the female patient the aetiocholanolone was about 4·5 times as much as the androsterone.

The observed increase in the ratio of aetiocholanolone to androsterone in the cases of prostatic disease might be produced by an increase in the absolute amount of aetiocholanolone, or by a decrease in the absolute amount of androsterone excreted, or by both simultaneously. The amounts of 3-(β)-hydroxyketosteroids, androsterone and aetiocholanolone excreted in 24-hours were calculated for the cases illustrated in Fig. 3. The results showed that the average daily excretion of aetiocholanolone in the two groups of cases (normal and prostatic disease) were approximately equal (2·0 and 1·9 mg. per day respectively), but that the androsterone excretion in the normal group (2·2 mg. per day) exceeded that of the prostatic group (1·25 mg. per day). These figures appear to support the conclusion that the actual difference between the two groups of subjects consists in a diminished output of androsterone by the prostatic group. This conclusion must, however, be regarded as provisional. Calculation of the daily excretions corresponding to peaks III, IV and V for all the cases shown in Table I indicates that there is a gradual decline with advancing age of each of these groups of substances, and hence the absolute excretions shown by the prostatic group (average age 65 years) should more properly be compared with a group of normal men of the same average age rather than with the slightly younger group (average age 59 years) shown in Fig. 3. Such a comparison will be possible when further normal males in the later decades have been examined.

In a study of α-ketosteroid excretion in patients with neoplastic disease Dobriner, Rhoads, Lieberman, Hill and Fieser (1944) found abnormal patterns in several cancerous conditions. In the one case of prostatic cancer reported in their paper there was a great excess of aetiocholanolone as compared with androsterone. They found a similar relation in a case of carcinoma of the larynx and a case of carcinoma of the breast. From the present results it appears, however, that such a relation is not necessarily characteristic of neoplastic disease, since the two cases of benign hyperplasia (Cases 19 and 20) show the same excess of aetiocholanolone; in one of them, indeed (Case 20), aetiocholanolone is present in greater excess than in any of the neoplastic cases.

Whatever the ultimate explanation may be of the relative increase of aetiocholanolone, it will be of great interest to investigate the effect of oestrogen therapy on the urinary 17-ketosteroid pattern in these cases of prostatic carcinoma. Material has been collected for this purpose and is being examined.

### SUMMARY.

Methods for the chromatographic separation and estimation of 17-ketosteroids in urine extracts are discussed, and a standard technique which yields reproducible analyses is described.

Urines from 19 normal males ranging in age from 3 to 73 years and urines from 2 cases of benign hyperplasia of the prostate and from 6 cases of carcinoma of this gland were examined by this method.

In normal males the amounts of androsterone and of aetiocholan-3-(α)-ol-17-one are approximately equal, but in prostatic disease the latter substance is present in relative excess.

The significance of this observation is discussed.

Generous grants to this Hospital by the British Empire Cancer Campaign have supported this work. We should also like to thank the Medical and Nursing Staff of St. Bartholomew's Hospital for helpful co-operation.

### REFERENCES.

Barton, D. H. R., and Klyne, W.—(1948) *Nature*, **162**, 493.

Brockmann, H., and Schodder, H.—(1941) *Berichte*, **74**, 73.

Callow, N. H.—(1939) *Biochem. J.*, **33**, 559.

Dingemanse, E., Huis in't Veld, L. G., and de Laat, B. M.—(1946) *J. clin. Endocrinol.*, **6**, 535.

*Idem*, Huis in't Veld, L. G., and Hartogh-Katz, S. L.—(1948*a*) *Nature*, **161**, 848.—(1948*b*) *Ibid.*, **162**, 492.

Dobriner, K., Lieberman, S., and Rhoads, C. P.—(1948) *J. biol. Chem.*, **172**, 241.

*Idem*, Rhoads, C. P., Lieberman, S., Hill, B. R., and Fieser, L. F.—(1944) *Science*, **99**, 494.

Huis in't Veld, L. G.—(1948) Thesis. Amsterdam.

Mason, H. L., and Kepler, E. J.—(1945) *J. biol. Chem.*, **160**, 255.—(1947) *Ibid.*, **167**, 73.

Patterson, J.—(1947) *Lancet*, **253**, 580.

Robinson, A. M.—(1948) *Brit. J. Cancer*, **2**, 13.

Wolfe, J. K., Fieser, L. F., and Friedgood, H. B.—(1941) *J. Amer. chem. Soc.*, **63**, 582.